# On the Surgical Treatment of Uterine Hemorrhage

**Published:** 1877-09

**Authors:** R. Stansbury Sutton

**Affiliations:** Pittsburg, Pa.


### ON THE SURGICAL TREATMENT OF UTERINE HEMORRHAGE.

By R. Stansbury Sutton, A. M., M. D., Pittsburg, Pa.

To the general practitioner of medicine, nothing is more alarming than excessive uterine hemorrhage. If it occur at, or prior to, the time of labor, from a placenta prævia, the practitioner recognizes the fact and acts in accordance with established principles. But if the hemorrhage occurs at the regular menstrual epochs, or between them, or if it is rarely absent, and a vaginal examination fails to reveal the cause, the medical man is often discouraged, from the fact that the cause of the hemorrhage is not apparent. In the last ten years it has been my fortune to meet with many cases of uterine hemorrhage. Fortunately they have all recovered. For the successful treatment of hemorrhage of this character, the first great remedy for the gaining of time, both for practitioner and patient, is a tampon. Tampons may be made of almost any material at hand. Soft linen or cotton rags are often employed. Some prefer sponges. These are often oiled before being packed into the vagina. Tampons of these substances thus prepared have been unsatisfactory to me. The tampon has soon become offensive, and required removal, having a foul odor, and adding to the discomfort and danger of the patient. I adopted, long ago, raw cotton as the best substance for the tampon, and pure glycerine as the best substance with which to smear it. The glycerine may be carbolized, but I do not find it necessary. The manner of making this tampon is as follows : unfold the roll of cotton and tear or cut off with scissors a piece 15 or 16 inches long, and as thick as your fore-arm. With the end of a stout string tie the cotton roll firmly 1½ or 2 inches from either end. With the long end of the string proceed as follows : carry it parallel with the cotton roll for two or three inches, and cast a loop around the roll, and thus continue with the string to the end of the roll, when the last loop should be secured with a knot. The tampon is now smeared with glycerine and is ready for use. How will you apply it? If convenient, Sims' speculum may be introduced. If not at hand, having soaped the index and middle finger of the left hand, pass one or both into the vagina, (the patient resting on her side,) turn the palmar surface of the fingers to the perineum and draw the latter *backwards* and *downwards*. All clots having been removed from the vagina, the tampon is now passed into the vagina, with the index finger of the right hand, along the posterior surface of the fingers supporting the perineum. If the tampon is too large to be entirely received, the redundant portion is pulled out of the string, which is left hanging from the vagina. In removing it, it will come away by traction just as regularly as it was introduced, and, in addition to being free from foul odor, will, if opened up, be found full of dry cotton. The advantages of this tampon are numerous; 1st. It is soft ; 2d. It absorbs blood very slowly; 3d. It is readily introduced; 4th. It is easily removed; 5th. It does not become foul; 6th. If congestion of the uterus is the cause of the bleeding a free drain of serum from the engorged vessels is set up, and soaks into the tampon, and it is therefore in such cases what no other tampon is, viz., *curative*. If, however, it is the choice to prefer some astringent in addition to the glycerine, my habit has been to apply to the mouth of the cervix a bit of cotton, well saturated with a solution of fluid extract of matico and glycerine, and then to apply the tampon in addition. Iron as a styptic is very effectual, as we all know, but it is objectionable for many obvious reasons in these cases. In addition to a good tampon in many cases, Barnes' water bags, sponge and laminaria tents are required to open the way to exploration of the uterine cavity.

In April, 1867, I was called to see Sarah, a colored servant. She was lying in a bed saturated with blood, almost pulseless, tongue pale and flabby. Her age was 48 years. For three or more years she had been occasionally the subject of these bleedings, but until now had been content with the thought that she was undergoing the change of life. An examination revealed a large fibroid polypus attached to the posterior lip of the uterus.

I ordered ergot and stimulants in liberal doses, and tamponed the vagina with soft linen cloths pushed in about the polypus. On the following morning I carried a silver wire, armed with Gooch's canula, around the neck of the polypus and strangulated it. Thirty-six hours later I severed the neck of the polypus below the wire with the scissors and removed it. Nourishing broths, and good nursing with tonics, gradually restored this woman's strength, and several months later I saw her in the city. Dr. E. S. Umstedter assisted me in the removal of the growth. A year after the operation, this woman developed cancer of the breast. She refused any operative procedure until the axillary glands became enlarged, after which I declined to operate. The operation, however, was done by another, and she died a week afterwards. Gooch's canula and wire have given place to the wire écraseru and galvano-caustic loop, both of which are among the improved surgical instruments.

In the latter part of November, 1871, I was called to see Miss Mc., aged 20. She was a girl of large frame, but emaciated and anemic. She had been confined to bed for several weeks, and her medical attendant had been stricken down with fatal disease. Her history was as follows: At 15 her menses appeared; until eighteen she menstruated regularly and was very stout and active. At this date she found the flow increasing, and her periods less frequent; at the end of a year she had periodical floodings. For two years she has passed almost half her time in bed, and is rarely free from some bleeding. On several occasions during the last year, tampons were required to save her life. On examination, the uterus is very much congested and heavy, the cervix is large and the os patulous; some blood is oozing from it.

The sound passes in $2\frac{7}{8}$ inches, and its introduction is followed by an increase in the flow. The feeling imparted through the sound is that of a rough, velvety surface. I passed into the patulous os a number of sponge tents; upon the removal of these I was able to pass the finger into the uterus, but from the uniform spongy feeling was led to believe that the uterus was lined with granulations, fungous, or, as I may say, polypoid in character. A large button of nitrate of silver was fused upon the end of a platinum probe, and while the mother held Sims' speculum, I cauterized the entire lining membrane of the uterus. When the slough separated, the hemorrhage was so great as to require a tampon to arrest it. Next in order during the winter of 1871-72, I tried styptics of every variety, and in March, 1872, hoped I was near the end of the trouble. The temporary relief was followed in May by a severe hemorrhage, which again put the patient to bed. I now again dilated the cervix, and explored the cavity of the uterus. Nothing new was elicited, but with Sims' curette I scraped out a number of small mucous polypi and rasped off the granular surface; to the now denuded surface I applied undiluted solution of the persulphate of iron. She recovered from this harsh treatment, and remained free from hemorrhages for a year. During this time her health improved very greatly, but she complained much of prolapsus, and was relieved with Hodge's pessary.

In November, 1874, I was called to see her again. She was in bed, and had just gotten over a prolonged and bloody menstruation. I again scraped out the uterus with the curette, this time finding several mucous polypi. On the third day afterwards, she left her bed on the first floor, in stocking feet and night-gown, and went to the third story for some article she wanted. The day following she had a chill, followed by cellulitis and abscess.

In due time the latter bursted, and she recovered by April, 1875. Having spent the summer in a country district, and taken a course of chalybeate waters, she married, Jan., 1876—thirteen months from the date of the second scraping with the curette. From the date of marriage until Dec. 30th, 1876, she was regular in every particular, when the menstruation ceased. Yesterday I was engaged to attend her in her approaching confinement.

In July, 1873, I was called to see Mrs. M. She was nursing a babe, about three or four months old. She was in bed, very pale and feeble. She said that blood had been "coming and going" from her womb for three weeks. A vaginal examination revealed no cause for it, and I introduced a sponge tent into the cervix. Ten or twelve hours afterwards, I removed the tent, and found in its meshes the remains of a mucous polypus. Believing this might be all, I waited. No further bleeding occurred.

Mrs. H., the mother of two children, called at my office, with her husband, in the summer of 1875, to consult me in regard to her health. Without doubt, her symptoms were those of pregnancy, and she admitted having felt motion, but said she was "unwell every three or four weeks." Her youngest child was 14 years old. She had morning sickness; the areolæ about the nipples were dark; the breasts were enlarged. She had frequent calls to empty the bladder, and leucorrhœa had annoyed her for six or eight weeks. Bimanual palpation with percussion, demonstrated an enlarged uterus, with the neck soft and apparently shortened. A speculum was now introduced. The vaginal mucous membrane was red, its rugae enlarged, and in the furrows a white opaque secretion, of creamy consistency, was apparent. But why did she bleed? Withdrawing the cylindrical speculum, I placed her upon her side, and inserted Sims' speculum, trusting her husband to hold it. I seized the anterior lip of the os with a small tenaculum, and, drawing down the cervix, observed a small pear-shaped polypus projecting from the os. It was now seized with a pair of fenestrated forceps, and its slender pedicle twisted off, when a second one was revealed and treated in the same way. These polypi were not glandular or mucous in character, but were abundantly supplied with cellular tissue and vessels. After this date, she did not have any further bleeding, and, at full term, I attended her in her confinement. Her child was a male, and weighed ten pounds.

Miss W. came under my care in the winter of 1871–72. The pelvis was filled with a sub-peritoneal fibroid tumor. Her menstruations were profuse and painful. After considerable trouble and time, I succeeded in dislodging the growth from the cavity of the pelvis. In the April following, the uterus measured in depth 5½ inches. Repeated sub-cutaneous injections of Squibb's extract of ergot, and the maintaining of the organ as free from flexion as possible, by appropriate pledgets of glycerized cotton, which not only assisted in supporting the organ, but drained it constantly, resulted in complete control of the hemorrhage. The uterus, a month ago, was but 2¾ inches deep, and menstruation was normal, notwithstanding the fact that the fibroid still exists, though much reduced in size.

Mrs. W., from a neighboring state, consulted me in April, 1875, on account of severe uterine hemorrhage, occurring irregularly. She was 50 years of age, and very large. The uterus was 7 inches deep, and attached to it was a very large sub-peritoneal fibroid tumor. She was ordered Squibb's fluid extract of ergot, and muriate of ammonium. A year later, she sent for me to see her in this city. She had been benefited by the former prescription, but still carried her large tumor. She declined to allow me to measure the uterus. A few weeks after, I was summoned to her in haste, but did not reach her for 12 hours, when I found her exhausted from loss of blood. A medical brother had tamponed her, and doubtless saved her life. I removed the tampon, cleaned out the vagina, and introduced a second tampon. Six or eight hours afterwards, the bleeding began again. The tampon was removed, the uterus found to be 5 inches deep, and losing blood slowly but constantly. A stream of cold water and vinegar was played against the cervix for a few minutes, followed by pure water. Some slight abatement was apparent. I now determined to stop it promptly, and, taking a long urethral syringe, bent its nozzle over the gas, to the proper curve, and filling it with a solution of persulphate of iron, carried it about 4 inches into the uterus and discharged its contents. Not another drop of blood appeared after its withdrawal. For several weeks she took salines and iron, with broths, milk and albuminous substances, and gained strength and flesh. Frequently, I have incidentally met her since, but although a year has passed, she has not had another hemorrhage.

In two cases of menorrhagia, caused by sub-peritoneal fibroids, the vaginal portion of the cervix has been slit up, bilaterally, with great relief to the patient. Whether tension, and consequently irritation, is thus relieved, I leave the reader to judge. The fact remains that it is a remedy in these cases.

In December, 1875, a lady from a Western State came to Pittsburgh on account of severe metrorrhagia, which had been of long duration. The uterus could be felt enlarged. The cervical canal was narrow and long. A sound proved the uterus three inches deep. The cervix was fully dilated with sponge tents, and a small submucous fibroid was enucleated. Her subsequent menstruations were normal.

Recently this patient writes me that her trouble is returning, and it is probable that the cause is another small fibroid.

In March, 1877, Mrs. D. consulted me on account of an irregularity in her menses. She did not complain so much of the loss of a great quantity of blood, as on account of the tardy disappearance of the flow, and the fact that it returned after apparent cessation. She had been married several years, and had no children. The cervix was very long and hard, and the os small. I slit up the cervix bilaterally to the vaginal attachment, and cut off with scissors the divided sides of the cervix. To one of these pieces I found attached a mucous polypus which probably accounted for the trouble. Since then she has menstruated naturally. The operation has left the os more open and made her more liable to pregnancy.

May, 1877.

